# The site-specific integration reaction of *Listeria* phage A118 integrase, a serine recombinase

**DOI:** 10.1186/1759-8753-4-2

**Published:** 2013-01-03

**Authors:** Sridhar Mandali, Gautam Dhar, Nuraly K Avliyakulov, Michael J Haykinson, Reid C Johnson

**Affiliations:** 1 Department of Biological Chemistry, David Geffen School of Medicine at UCLA, Los Angeles, CA, 90095-1737, USA; 2 Present address: Department of Obstetrics and Gynecology, David Geffen School of Medicine at UCLA, Los Angeles, CA, 90095, USA; 3 Molecular Biology Institute, University of California, Los Angeles, Los Angeles, CA, 90095, USA

**Keywords:** Serine recombinases, Phage integrases, *In vitro* recombination, Domain structure, Recombination (*att*) site determinants and specificity

## Abstract

**Background:**

A large subfamily of serine recombinases contains long polypeptide segments appended to the C-terminal end of the conserved catalytic domain. Members of this subfamily often function as phage integrases but also mediate transposition and regulate terminal differentiation processes in eubacteria. Although a few members of this subfamily have been studied in purified *in vitro* systems, key mechanistic aspects of reactions promoted by these recombinases remain to be determined, particularly with respect to the functions of the large C-terminal domain.

**Results:**

We have developed and characterized a robust *in vitro* recombination reaction by the *Listeria* phage A118 integrase, a member of the subfamily of serine recombinases containing a large C-terminal domain. The reaction occurs in a simple buffered salt solution and exhibits a modest stimulation by divalent cations or spermidine and DNA supercoiling. Recombination with purified A118 integrase is unidirectional, being efficient only between *attP* and *attB* DNA sites to either join separate DNA molecules (intermolecular recombination) or to generate deletions or inversions depending on the relative orientation of *att* sites in cis (intramolecular recombination). The minimal *attP* site is 50 bp but requires only 44 bp of base sequence information, whereas the minimal *attB* site is 42 bp and requires 38 bp of base sequence information. DNA exchange occurs between the central 2 bp of *attP* and *attB*. Identity between these two base pairs is required for recombination, and they solely determine the orientation of recombination sites. The integrase dimer binds efficiently to full *att* sites, including the *attL* and *attR* integration products, but poorly and differentially to each half-site. The large C-terminal domain can be separated from the N-terminal catalytic by partial proteolysis and mediates non-cooperative DNA binding to *att* sites.

**Conclusions:**

The basic properties of the phage A118 integrase reaction and its substrate requirements have been elucidated. A118 integrase thus joins the handful of biochemically characterized serine integrases that are serving as models for mechanistic studies on this important class of recombinases. Information reported here will also be useful in exploiting this recombinase for genetic engineering.

## Background

**Figure 1:**
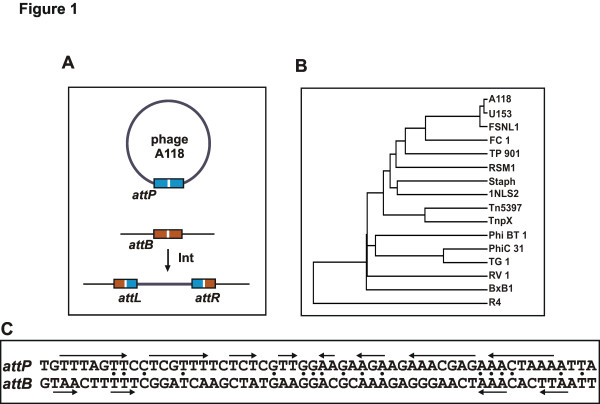
**Phage A118 integration reaction and the subfamily of serine recombinases with large C-terminal domains. (A)** Schematic representation of phage A118 integrative recombination, where *attP* on the phage genome recombines with *attB* within *comK* in the *Listeria monocytogenes* chromosome to form the two junction recombination sites *attL* and *attR*. **(B)** Phylogenetic tree of representative serine recombinases with large C-terminal domains (ClustalW2). **(C)** Sequence (56 bp) surrounding the A118 *attP* and *attB* loci. Palindromic sequences within each *att* site are denoted with arrows, and identical nucleotides between the two recombination sites are marked with dots. We show in this work that the central GG nucleotides define the crossover sites.

Programmed DNA rearrangements mediated by site-specific recombinases mediate a diversity of biological reactions. The *Listeria* phage A118 integrase, the subject of this report, catalyzes integration and excision of the viral genome into and out of a specific locus within the bacterial host chromosome (Figure 1A) [1]. Other site-specific recombination (SSR) reactions regulate expression of cell surface proteins, promote the transfer of virulence and antibiotic resistance genes, maintain monomeric circular chromosomes for faithful segregation, or resolve transposition intermediates [2]. Well-characterized SSR systems have been exploited for genetic engineering purposes and have thus greatly added to the molecular geneticists’ toolbox [3-6]. SSR reactions are often intricately controlled and studies of their regulation have revealed new concepts for nucleoprotein complex assembly as well as DNA enzymology.

Most conservative SSR reactions, where there is no net gain or loss of DNA sequences, fall into two enzymatic families that promote DNA exchange by completely different biochemical mechanisms [7]. Members of the tyrosine recombinase family generate single-strand DNA breaks through the nucleophilic attack of a tyrosine hydroxyl. Two sequential single-strand DNA exchange steps with the formation of a Holliday structure intermediate are required to complete recombination [8,9]. By contrast, members of the serine recombinase family generate double strand breaks in DNA through the concerted action of a pair of active site serine residues within the dimeric enzyme. DNA exchange occurs by translocation of subunits that are covalently linked to the cleaved DNA ends within the recombination complex in a reaction known as subunit rotation [10-15].

A118 integrase is a member of the serine recombinase family [16]. Members of this family all share a fairly well conserved 100 to 120 residue catalytic domain followed by a long α-helix (referred to as helix E from resolvase structures), which forms much of the interface between dimer and tetramer forms of the enzyme [7]. Additional domains are appended on the N-terminal or more often C-terminal sides of the catalytic and oligomerization domain. The most intensively studied subfamily of serine recombinases, known as the resolvases and DNA invertases, have a relatively short (typically <60 residue) DNA binding domain at their C-terminal end [17,18]. Well-developed models for orientation-specific DNA site synapsis and DNA exchange have been described for these enzymes that are supported by atomic structures and extensive biochemistry [10,11,15,19-22].

Another subfamily of serine recombinases has much longer C-terminal segments that typically extend 300 to 500 residues [23]. A few members of this group have been investigated in detail, such as the integrases from phages ϕC31, Bxb1, and ϕRv1, and the TnpX transposase from *Clostridia*[24-27]. The relationship of the integrase sequences from phages A118 and its close relative U153, together with other representatives of this subfamily, is shown in Figure 1B. A feature in common with most of the large C-terminal domains is a relatively centrally located segment with four conserved cysteines [23]; in A118 integrase over one-third of the 41 residues in this segment are also arginine, lysine or histidines. The functional roles of the large C-terminal domain in any of the recombinases remain poorly understood, except that they have been found to specify DNA binding and control aspects of site synapsis in several systems [24,28,29]. There is currently no structural information on the large C-terminal domain from any member of the subfamily.

The phage A118 integrase was first described by Calendar and coworkers as part of their analysis of the phage genome sequence [1]. They showed that both A118 and the related U153 phage site-specifically integrate into a *Listeria monocytogenes* chromosomal locus called *attB* located within the coding region of a gene resembling *Bacillus subtilis comK*, whose product encodes a transcriptional activator of genes involved in DNA uptake [30]. The sequences surrounding the A118 *attP* recombination site and *attB* are poorly related, and the *attB* region exhibits remarkably little symmetry (Figure 1C). Calos and coworkers reported that the A118 integrase together with DNA fragments containing *attP* and *attB* can promote recombination in *Escherichia coli* and mammalian cells, thereby demonstrating its potential for genetic engineering [31].

In this study we characterize the basic biochemical properties of the A118 *attP*×*attB* integration reaction using purified integrase. We define the minimal recombination sites and crossover sites and study the binding properties of integrase to the *att* sites. The properties of the A118 integrase are compared with those of other characterized members of the subfamily of serine recombinases containing large C-terminal domains.

## Results

### Purification and partial proteolysis of A118 integrase

**Figure 2:**
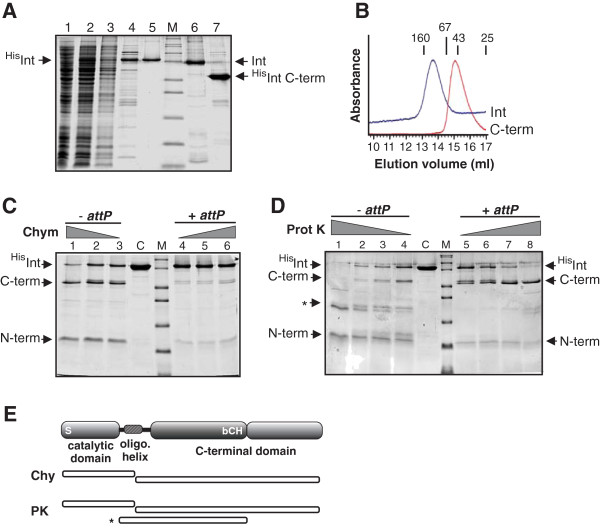
**Purification and partial proteolysis of A118 integrase. (A)** SDS-PAGE of A118 integrase purification. Lanes 1 to 3, uninduced, induced, and extract after clearing spin, respectively. Lane 4, N-terminally His-tagged integrase after Ni-NTA, and then heparin-Sepharose chromatography (lane 5). Lane 6, native integrase after heparin-Sepharose chromatography, and (lane 7) His-tagged C-terminal domain (residues 158 to 452) after Ni-NTA chromatography. Lane M, molecular weight (MW) markers are 6, 14.4, 21.5, 31, 36.5, 55.4, 66.3, 97.4, 116.3, and 200 kDa. **(B)** Size exclusion chromatograms (Superdex 200) of full-length ^His^Int (calculated dimer MW = 110,684) and ^His^Int^158-452^ (C-term, calculated monomer MW = 37,438). Size standards are chymotrypsin (25 kDa), ovalbumin (43 kDa), BSA (67 kDa), and γ-globulin 160 (kDa). **(C** and **D)** Partial proteolysis of A118 integrase by chymotrypsin and proteinase K, respectively. Integrase (3 μg) was digested with 20, 40, and 60 ng chymotrypsin or 50, 75, 100 and 125 ng proteinase K for 10 min at 23°C in the absence or presence of twofold molar excess of 50 bp *attP* oligonucleotides, as designated. The products were displayed on a 15% SDS-PAGE gel. Lane C, no protease control; MW markers (lane M) range from 6 to 66.3 kDa, as in (A). **(E)** Schematic representation of the A118 integrase domain structure based on proteolysis experiments and secondary structure prediction programs. Mutagenesis experiments have shown that the putative active site serine (S) at residue 10 within the catalytic domain is required for recombination. The long putative oligomerization helix is analogous to α-helix E in resolvase structures. bCH, a basic (11 arginines and lysines) segment between residues 274 and 314 containing four cysteines and four histidines. Chymotrypsin and proteinase K fragments identified by mass spectrometry are represented below (see text).

Highly purified preparations of N-terminally histidine tagged A118 integrase were obtained by chromatography through Ni-NTA followed by heparin-Sepharose (Figure 2A, lanes 1 to 5). The activity and tested properties of the His-tagged integrase were indistinguishable from partially purified untagged full-length integrase obtained by heparin-Sepharose chromatography (Figure 2A, lane 6; and data not shown). Because the N-terminal tag has no apparent effect on activity, we have employed the more highly purified tagged protein for the studies in this paper. A C-terminal domain fragment beginning at residue 158 was also expressed and partially purified by means of its N-terminal histidine tag (Figure 2A, lane 7). This polypeptide exhibits no catalytic activity but was used for DNA binding studies. Size exclusion chromatography demonstrated that full-length integrase is a dimer in solution, whereas the recombinant C-terminal domain fragment was monomeric (Figure 2B).

Integrase was subjected to limited proteolysis to probe its domain structure. Digestion with chymotrypsin generated two dominant products of approximately 40 and 17 kDa (Figure 2C, lanes 1 to 3). Matrix-assisted laser desorption/ionization time of flight/time of flight (MALDI-TOF/TOF) mass spectrometry after trypsin digestion of the 40 kDa band generated tryptic peptides extending from integrase residues 143 to 427 and of the 17 kDa band from residues 9 to 129. In a separate experiment, tryptic peptide sequences from the 40 kDa band began at residue 136. Chymotrypsin cleavage at 130 would generate calculated molecular weight products of 16,786 (1 to 129 plus His tag) and 38,400 (130 to 452), corresponding to the sizes measured by SDS-PAGE. These data indicate a major chymotrypsin-sensitive site between residues 130 and 136 that separates the small N-terminal catalytic domain from the large C-terminal domain (Figure 2E).

Increasing proteinase K digestion generated three major products migrating at approximately 40 kDa, 27 to 25 kDa, and 17 to 15 kDa (Figure 2D). Trypsin digestion and mass spectrometry of the 40 kDa fragment gave the same C-terminal tryptic peptides as the large fragment from the chymotrypsin experiment. The 15 kDa fragment surviving proteinase K digestion gave four tryptic peptides between residues 9 and 115 (an additional low-intensity peptide corresponding to residues 233 to 246 was also identified). The 27.5 kDa fragment (Figure 2D, asterisk) produced seven tryptic peptides between residues 96 and 331 (calculated molecular weight = 27,823). We interpret this fragment to represent a cleavage at the C-terminal end of the catalytic domain and one shortly after the cysteine-histidine rich segment within the C-terminal domain (Figure 2E).

The above proteolysis reactions were all performed without DNA present. We also performed identical experiments in the presence of excess *attP* DNA. As shown in Figure 2C (lanes 4 to 6), integrase was much more resistant to chymotrypsin cleavage when bound to *attP*; only a trace of the N-terminal and C-terminal domain products are evident. Cleavage by proteinase K generating the N-terminal and C-terminal domain products remained relatively robust in the presence of *attP* DNA, but formation of the 27.5 kDa product was strikingly inhibited (Figure 2D, lanes 5 to 8). These results provide evidence for conformational differences between free and DNA-bound forms of integrase that influence protease accessibility or reactivity.

### Intermolecular and intramolecular recombination by A118 integrase

**Figure 3:**
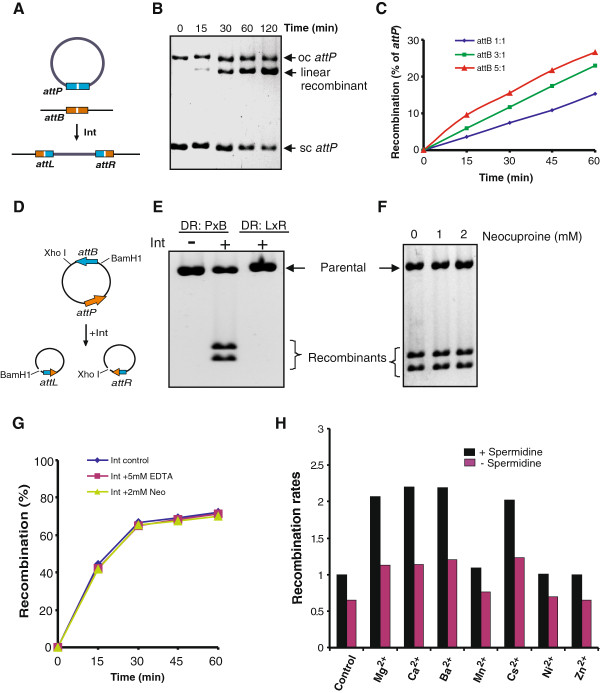
***In vitro *recombination by A118 integrase. (A)** Integrative recombination between a 4.8 kb supercoiled plasmid containing *attP* (pRJ2214) and a 100 bp *attB* fragment generates a 4.9 kb linear product. **(B)** Agarose gel showing a time course of intermolecular recombination between supercoiled *attP* and linear *attB* substrates. **(C)** Titration of increasing amounts of the *attB* fragment relative to the supercoiled *attP* plasmid on rates of intermolecular recombination. Molar ratios of *attB* to *attP* (set at 1) are given. **(D)** Intramolecular deletion reaction between *attP* and *attB* on pBCPB-A1+ generates 3.5 and 3.9 kb deletion products that are linearized after digestion with *Bam*HI and *Xho*I, respectively. **(E)** Deletion reactions between *attP* and *attB* (pBCPB-A1+) or between *attL* and *attR* (pRJ2913; no recombinant products formed). Integrase reactions were for 40 minutes. **(F)** Deletion reactions (*attP*×*attB*) performed in the absence or presence of the strong metal chelator neocuproine. Integrase reactions were for 40 minutes. **(G)** Recombination rates in the presence of chelators. Deletion reactions were performed using integrase from storage buffer with 0.1 mM ethylenediamine tetraacetic acid (control, dark blue) or pre-incubated at 4°C overnight with 50 mM EDTA (red) or 20 mM neocuproine (green) in buffer without added metal. Final concentrations of EDTA and neocuproine in the reactions were 5 and 2 mM, respectively. **(H)** Recombination rates in reactions supplemented with metals and polyamines. Supplements were added to 10-minute deletion reactions with or without 5 mM spermidine. Supplements (MgCl_2_, CaCl_2_, BaCl_2_, MnCl_2_, CsCl, and NiCl_2_) were at 10 mM except for ZnSO_4_, which was at 1 mM because higher amounts were inhibitory; FeCl_2_, CoCl_2_, CuCl_2_ at 1 mM were inhibitory. Recombination rates (% deletions in 10 minutes, average of at least three experiments) are given relative to the unsupplemented reaction (control) in the presence of spermidine, which was set to 1.

Initial experiments testing the activity of purified integrase employed a supercoiled plasmid (pRJ2214) containing *attP* together with a 100 bp fragment containing *attB* in a reaction analogous to phage integration (Figure 3A). As shown in Figure 3B, linear recombinant products, which were confirmed using radiolabeled *attB* fragments (data not shown), increased with incubation time. Reaction rates improved with increasing ratios of *attB* to *attP*, with maximum rates achieved at a 5:1 molar ratio of linear *attB* to supercoiled *attP* (0.03 pmol) with 1 pmol Int (Figure 3C). Subsequent intermolecular reactions employed a 3:1 ratio of short linear to supercoiled plasmid substrates.

We asked whether integrase could catalyze an intramolecular *attP*×*attB* deletion reaction as shown in Figure 3D. As shown in Figure 3E (lane 2), Int was efficient at promoting intramolecular deletions between *attP* and *attB* as revealed by the formation of the two deletion circles, which were each subsequently linearized by restriction enzymes. However, no deletion products were detected in similar intramolecular reactions between *attL* and *attR*, the substrates for the excision reaction by phage A118 (Figure 3E, lane 3). Reactions that included *L*. *monocytogenes* or *E*. *coli* extracts or purified HU also did not generate detectable *attL*×*attR* deletion products (data not shown). A phage-encoded directionality factor is therefore probably required for the *attL*×*attR* excision reaction.

### *In vitro* reaction conditions: role of metals, divalent cations, and polyamines

Reactions were initially performed in 20 mM Tris buffer (pH 7.5), 100 mM NaCl, 5 mM spermidine, 5 mM dithiothreitol (DTT), 100 μg/ml BSA, and 5% glycerol. We individually varied components to evaluate parameters and optimize the *in vitro* reaction rates using the intramolecular *attP*×*attB* deletion reaction. The reaction was optimal over a broad range of NaCl concentration from 50 to 200 mM, but KCl or K glutamate gave up to 20 to 30% greater reaction than NaCl. Perhaps surprisingly, given the cysteine-rich domain, the reaction was not affected by redox conditions; the absence or presence of up to 10 mM DTT or 5 mM oxidized glutathione had little effect on deletion rates. The presence of 5%, 10%, or 15% glycerol gave 30 to 50% enhancement and substituting glycerol with ethylene glycol gave 55 to 70% enhancement. The optimal temperature for the reaction was 30°C, which was used throughout this work. Experiments employing extracts or purified HU from *L*. *monocytogenes* provided no evidence for a stimulatory co-factor.

ϕC31 integrase was recently reported to contain zinc, which is believed to be associated with its cysteine-rich motif, and the binding of zinc was shown to be functionally important for binding to DNA and recombination [32]. However, other serine integrases promote recombination efficiently *in vitro* without an apparent requirement for zinc [27,33-35]. Our experiments provide no evidence for a functional role of zinc for A118 integrase. Addition of up to 10 mM ethylenediamine tetraacetic acid (EDTA), 8 mM cyclohexylenedinitrilotetraacetic acid, 10 mM ethylene glycol tetraacetic acid, or 2 mM neocuproine (2,9-dimethyl-1,10-phenanthroline) to reactions containing spermidine had no inhibitory effect on *attP*×*attB* deletion rates. In the experiment shown in Figure 3G, A118 integrase was incubated overnight at 4°C with 50 mM EDTA or 20 mM neocuproine and the treated enzyme was added to the reaction to give a final concentration of 5 mM EDTA or 2 mM neocuproine. No differences with the untreated control in the rate of product accumulation were evident. Addition of up to 1 mM zinc to reactions without chelators present had no stimulatory effect. Integrase was also expressed in Luria-Bertani broth (LB) supplemented with a mixture of inorganic micronutrients including 0.5 mM zinc [36] and purified by Ni-affinity chromatography with zinc in all buffers including the storage buffer. The yield and activity of this preparation was indistinguishable from standard integrase preparations.

The effects of divalent cations and metals and their relationship with spermidine on the A118 integrase reaction are summarized in Figure 3H. The presence of spermidine resulted in a 50% increase in reaction rates. Addition of Mg^2+^, Ca^2+^, Ba^2+^, or Cs^+^ in the absence or presence of spermidine gave up to twofold increases in rates but Mn^2+^, Ni^2+^, or Zn^2+^ had no measurable effects. Int binding to *attP* DNA fragments by gel mobility shift assays showed no differences in binding affinities when EDTA, MgCl_2_, or ZnSO_4_ were included in the binding reactions and electrophoresis buffers (data not shown). These data provide no evidence for an important role for a metal in DNA binding or catalysis by the A118 integrase. The small enhancements of the reaction by additives such as Mg^2+^, Ca^2+^, Ba^2+^, and spermidine may be due to global effects on DNA structure [37,38].

### DNA substrate specificity: *att* sites and DNA topology

**Figure 4:**
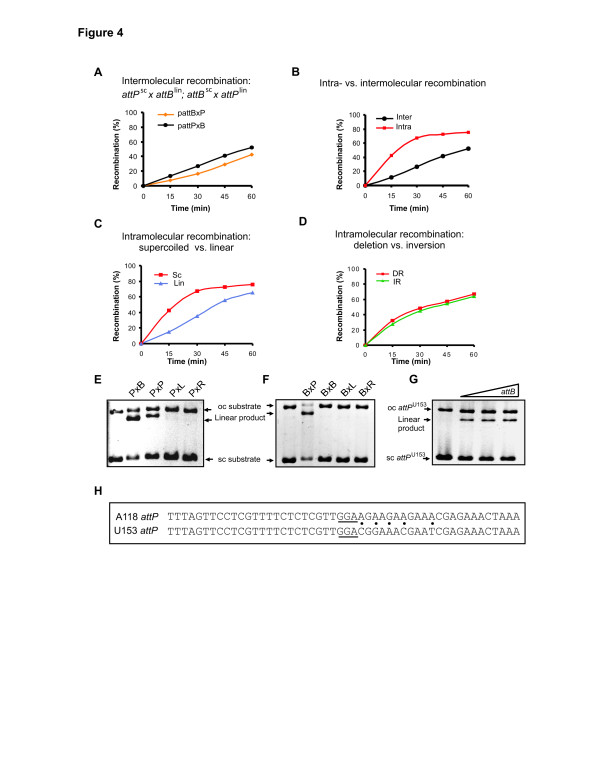
**Substrate topology and *att *site specificity. (A)** Comparison of intermolecular integration reactions between supercoiled *attP* (pRJ2214) and linear *attB* (100 bp, threefold molar excess over *attP*) and between supercoiled *attB* (pRJ2215) and linear *attP* (100 bp, threefold molar excess over *attB*) substrates. **(B)** Comparison of intramolecular *attP×attB* deletion reactions (pBCPB-A1+) and intermolecular integration reactions between supercoiled *attP* (pRJ2214) and linear *attB*. **(C)** Comparison of intramolecular *attP*×*attB* deletion reactions on supercoiled and linear pBCPB-A1+. **(D)** Comparison of intramolecular *attP*×*attB* deletion (pBCPB-A1+) and inversion (pRJ2799) reactions. These two supercoiled substrates are identical except for the relative orientation of *att* sites. **(E)** Intermolecular integration reactions (40 minutes) between supercoiled *attP* (pRJ2214) and 100 bp linear fragments containing different *att* sites at threefold molar excess over *attP*. **(F)** Intermolecular integration reactions (40 minutes) between supercoiled *attB* (pRJ2214) and 100 bp linear fragments containing different *att* sites at threefold molar excess over *attB*. **(G)** Intermolecular integration reactions between supercoiled *attP* from phage U153 (pRJ2289) and 100 bp linear fragments containing the *attB* site that is used by both phages. **(H)** Sequence of the A118 and U153 *attP* sites. Non-identities on the P’ sides are highlighted with dots, and the three central nucleotides in common with *attB* are underlined.

A series of experiments were performed to assess the effects of substrate topology and *att* site location on the A118 integrase reaction. In general, relatively small differences in reaction rates were observed comparing intermolecular versus intramolecular *attP*×*attB* reactions on substrates with different topologies. Intermolecular integration reactions were reproducibly slightly more efficient when *attP* was located on a supercoiled plasmid and *attB* was located on a 100 bp linear fragment than when *attB* was on the plasmid and *attP* was on the linear fragment (Figure 4A). A comparison of intermolecular reactions between two supercoiled plasmids versus one supercoiled (*attP* or *attB*) plasmid and one linear (*attB* or *attP*) plasmid showed little differences in rates or yields, but recombination rates between two linear plasmids were reduced by about 50% (data not shown). The intramolecular *attP*×*attB* deletion reaction exhibited about threefold greater reactions rates than the intermolecular supercoiled *attP*×linear *attB* reaction (Figure 4B), presumably because of a greater frequency of *att* site collisions when located in cis [39]. DNA supercoiling enhanced the intramolecular deletion reaction by about threefold (Figure 4C), which can also be rationalized by an increase in *att* site collisions generating productive synapses. There was no measurable difference in deletion or inversion rates between *attP* and *attB* sites separated by 3 kb in a direct or inverted orientation, respectively, on a supercoiled plasmid (Figure 4D).

Although integrase alone cannot catalyze *attL*×*attR* recombination (Figure 3E), we asked whether recombination could occur between *attP* or *attB* and each of the other *att* sites (Figure 4E,F). Recombination can occur between two *attP* sites, but the rate is <5% that of the *attP*×*attB* reaction. By contrast, two *attB* sites do not support detectable recombination and no reactions are observed between *attL* or *attR* and *attP* or *attB*. We conclude that the A118 integrase exhibits high specificity for the different *att* sites with only *attP*×*attB* functioning effectively.

A118 and U153 integrases from *L*. *monocytogenes* phages are very similar in sequence, sharing 95% amino acid identity (Figure 1B). Moreover, *in vivo* experiments have shown that phages A118 and U153 lysogenize at the same *attB* locus and that their integrases complement each other [30,31]. The two *attP* loci are identical within their left half-sites but have five sequence differences on their right half-sites (Figure 4H). We find that the A118 integrase will catalyze a low level of integration *in vitro* between a supercoiled plasmid containing *attP*^*U153*^ and linear *attB* but that initial rates and final product yields are threefold to fourfold less than with *attP*^*A118*^ (Figure 4G; and data not shown). A118 integrase binds to *attP*^*U153*^ with similar affinity as *attP*^*A118*^ (data not shown), so the reduced recombination efficiency with *attP*^*U153*^ cannot be explained by inefficient binding of the integrase to its noncognate *attP* site.

### Binding of A118 integrase to *att* sites

**Figure 5:**
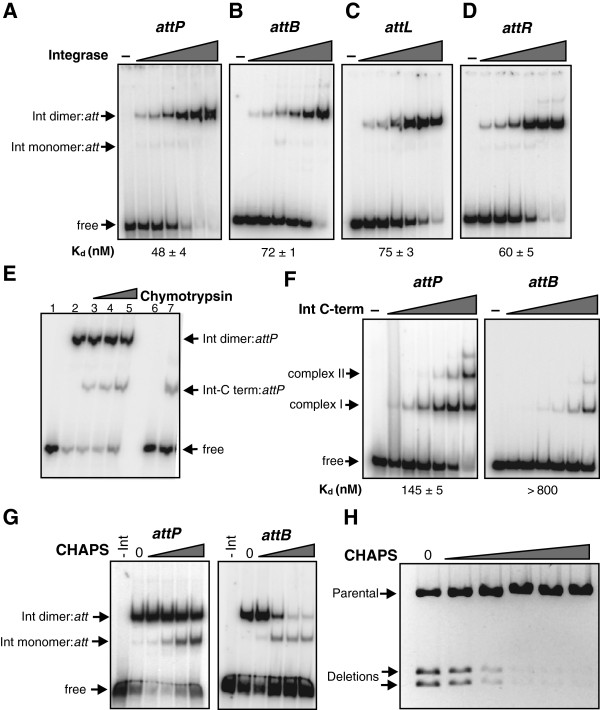
**A118 integrase binding to different *att *sites.** Gel mobility shift assays with increasing amounts of integrase and ^32^P-labeled 100 bp fragments containing **(A)***attP*, **(B)***attB*, **(C)***attL*, and **(D)***attR*, respectively. Integrase concentrations were 0, 5, 10, 30, 90, 270, and 810 nM. Assignments of monomer and dimer DNA complexes were based on their migrations relative to complexes formed by the integrase C-terminal domain (for example, **(E)**,**(F)**) and in CHAPS titration experiments (for example, **(G)**). Apparent dissociation constants (K_d_ values) for the dimer complexes (mean and standard deviation from three experiments) are given below each panel. **(E)** Gel mobility shift assay employing integrase subjected to partial proteolysis with chymotrypsin (0, 20, 40, and 60 ng for 10 minutes) and ^32^P-labeled *attP* fragments (lanes 2 to 5). The chymotrypsin-digested integrase samples used for this experiment appeared nearly identical by SDS gel electrophoresis to those in Figure 2C, lanes 1 to 3. Lanes 1 and 6, controls without protein added; lane 7, reaction with the purified recombinant C-terminal integrase domain (30 nM). **(F)** Gel mobility shift assays with increasing amounts of the recombinant integrase C-terminal domain and ^32^P-labeled 100 bp fragments containing *attP* or *attB*. Integrase C-terminal domain concentrations were 0, 5, 10, 30, 90, 270, and 810 nM. **(G)** Effect of CHAPS on integrase binding to *att* sites. Integrase (90 nM) was incubated with *attB* and *attP* in the presence of 0, 5, 10, 15, and 20 mM CHAPS. No integrase was added to the left lanes of each panel. **(H)** Effect of CHAPS on integrase recombination activity. Intramolecular *attP*×*attB* deletion reactions (15 minutes) were performed in the presence of 0, 2, 4, 6, 8, and 10 mM CHAPS.

Binding of A118 integrase to ^32^P-labeled 100 bp fragments containing *attP*, *attB*, *attL*, and *attR* was evaluated using gel mobility shift assays. As shown in Figure 5A, increasing amounts of integrase were incubated with the *attP* probe, and the reaction mixture was subjected to electrophoresis in native polyacrylamide gels. The dominant complex contains a dimer of integrase with only a trace of a complex containing an integrase monomer detectable. The binding properties of *attB*, as well as *attL* and *attR*, are similar to *attP* with apparent K_d_ values for all sites ranging from 48 to 75 nM (Figure 5A). Integrase thus binds efficiently to *attL* and *attR*, even though reactions employing these substrates are not productive for recombination.

A118 integrase that had been incubated with increasing amounts of chymotrypsin under conditions similar to those in Figure 2C was employed in gel mobility shift assays with *attP* in Figure 5E (lanes 3 to 5). The partially proteolyzed Int generated a single additional complex of intermediate mobility with that of the full-length dimer complex (lane 2). The migration of this complex corresponds to the migration of the DNA complex formed with a recombinant preparation of the C-terminal domain from residues 158 to 452 (Figure 5E, lane 7; see also Figure 2A, lane 7). The C-terminal domain is therefore sufficient to specify DNA binding activity.

The binding properties of the isolated C-terminal domain were then evaluated (Figure 5F). Unlike full-length integrase, the C-terminal domain predominantly generates Complex I on *attP* and *attB* that would correspond to the binding of a single protomer. Complex II, which forms inefficiently on both substrates, probably contains two protomers. The C-terminal domain binds *attP* much more efficiently than *attB*. This contrasts with full-length integrase, which exhibits only 1.5-fold better binding to *attP* over *attB* (Figure 5A,B). There is no evidence for cooperative binding by the C-terminal domain, unlike the full-length integrase that binds as a highly cooperative dimer (elaborated further below).

The zwitterionic detergent CHAPS has been shown to specifically destabilize the Hin recombinase dimer [40,41]. We asked how CHAPS affects A118 integrase binding to *attP* and *attB*. As shown in Figure 5G, the presence of CHAPS around its critical micelle concentration enhances monomeric binding of full-length integrase to both *attP* and *attB*. Integrase binding to *attB* is particularly sensitive to CHAPS as dimer binding is strongly inhibited and the monomeric form predominates above 10 mM detergent. Likewise, CHAPS above 4 mM has a strong inhibitory effect on recombination activity (Figure 5H). These data are consistent with CHAPS destabilizing the oligomerization interface of the A118 integrase, which disturbs binding cooperativity and inhibits formation of a recombinationally active synaptic complex. The nonionic detergent Triton X-100 has no significant effect on integrase binding or recombination at levels above its critical micelle concentration (data not shown).

### Integrase binding to half-*att* sites

**Figure 6:**
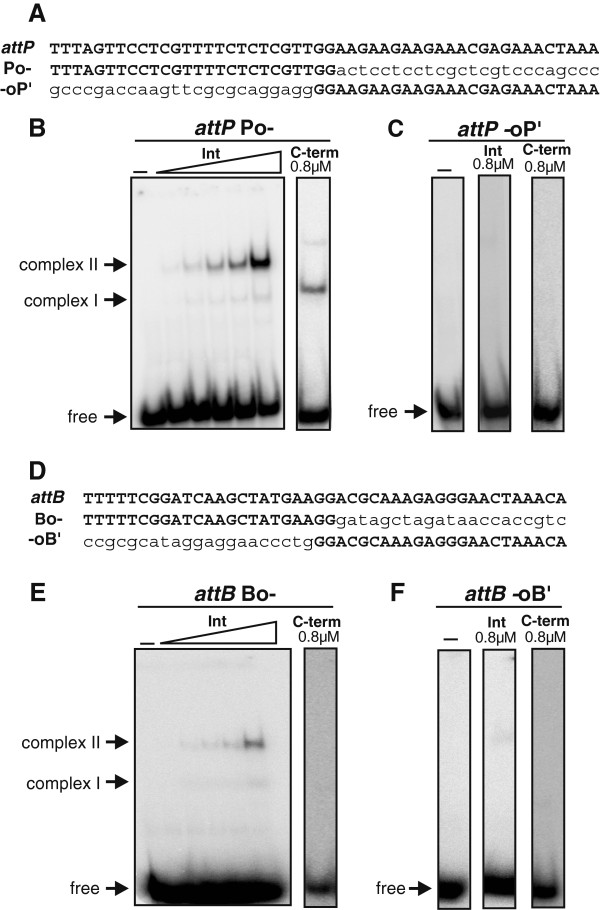
**Integrase binding to half**-***att *sites. (A)** Sequences of *attP* half-sites (Po- and -oP’). Lowercase letters designate non-*att* sequences on the duplex probes. **(B)** Gel mobility shift assay with the Po- half-site. Left panel contains increasing concentrations of integrase (0, 10, 30, 90, 270, and 810 nM), and right panel shows binding with 810 nM of the C-terminal integrase domain. **(C)** Gel mobility shift assay with the -oP’ half-site using 810 nM of integrase or the C-terminal domain. **(D)** Sequences of *attB* half-sites (Bo- and -oB’). **(E)** Gel mobility shift assay with the Bo- half-site. Left panel contains increasing concentrations of integrase (0, 10, 30, 90, 270 and 810 nM), and right panel shows binding with 810 nM of the C-terminal domain. **(F)** Gel mobility shift assay with the -oB’ half-site using 810 nM of integrase and the C-terminal domain.

The differential effects of CHAPS on *attP* and *attB* by full-length Int, combined with the avid binding of a single C-terminal domain protomer to *attP* relative to *attB*, suggest that Int does not bind to the two *att* sites in an equivalent manner (Figure 5). To evaluate this further, binding by the full-length Int and the C-terminal domain to each of the four *att* half-sites (see below) was probed. The *attP* sequence is designated PoP’ and the *attB* sequence is designated BoB’ to represent the left and right half-sites and central crossover (o) regions as written in Figure 1B. Full-length Int bound predominantly as a dimer (K_d_ ~ 1 μM) and the C-terminal domain exhibited weak monomer binding to the *attP* Po half-site, but no detectable binding to the oP’ half-site (Figure 6A,B,C). Full-length Int exhibited weak dimer binding (K_d_ > 1 μM) to *attB* Bo, but again no binding was detectable to the right oB’ half-site (Figure 6D,E,F). Dimeric binding to the left half-sites, even in the case for an *attP* probe where no DNA was present over the right half-site (data not shown), confirms the strong dimer cooperativity with full-length Int. These results also show that the two half-sites of *attP* and *attB* are functionally distinct since neither the P’ nor B’ half-sites exhibit detectable binding.

### The minimal *attP* and *attB* sequence

**Figure 7:**
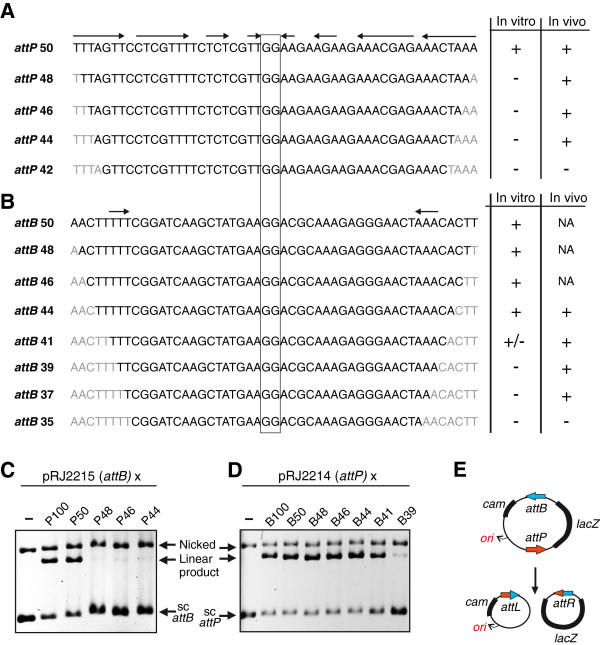
**Determination of minimal *attB *and *attP *lengths required for efficient recombination. (A)** Sequence of the resected *attP* substrates and their abilities to support *in vitro* intermolecular integration (for example **(C)**) or *in vivo* intramolecular deletion (for example **(E)**). Symmetry-related segments denoted above the *attP* and *attB* sequences, and the GG dinucleotide crossover segments (see Figure 8) are boxed. *In vitro* data: (+), >60% recombinants; (−), little–no detectable products in 40-minute reactions. *In vivo* data: (+), >90% white colonies (deletion products); (−), <10% white colonies. Sequences in light grey are absent in the *in vitro* reactions and replaced with different nucleotides in the *in vivo* reactions. **(B)** Sequence of the resected *attB* substrates and their abilities to support *in vitro* intermolecular integration (for example **(D)**) or *in vivo* intramolecular deletion. Activities are designated as in **(A)**; *in vitro* reaction rates for *attB* 41 (+/−) were about 15% of those of the longer substrates. NA, not analyzed. **(C), (D)** Representative gels showing *in vitro* intermolecular integration reactions (40 minutes) between supercoiled *attB* (pRJ2215) and resected *attP* fragments **(C)** or between supercoiled *attP* (pRJ2214) and resected *attB* fragments. **(E)** Illustration of the *in vivo attP*×*attB* deletion reaction with pBCPB-A1+ [31]. Deletion generates two circular products that may be topologically linked, but only the *lacZ*^–^Cam^r^ product containing the replication origin will be maintained.

The minimal sequence requirements for *attP* and *attB* function were determined both *in vitro* and *in vivo*. The minimal sequence length for *attP* was determined *in vitro* by intermolecular integration reactions between supercoiled pRJ2215 containing a 100 bp *attB* segment and a series of duplex oligonucleotides of decreasing lengths representing the *attP* sequence (Figure 7A,C). A synthetic duplex of 50 bp covering the imperfect palindromic sequence surrounding the *attP* crossover point supported recombination with similar efficiency to a 100 bp *attP* fragment. However, a 48 bp duplex that eliminated the outer base pairs exhibited no detectable recombination *in vitro*.

Recombination proficiency of the resected *attP* sites *in vivo* was evaluated by intramolecular deletion formation in *E*. *coli* using plasmids derived from pBCPB-A1+ [31]. The *attP* region was replaced with the resected *attP* segments and transformed into a *recA* Δ*lac* strain containing a compatible plasmid (pIntA1) expressing integrase. Deletion removes the *lacZ* gene between *attP* and *attB*, resulting in white colonies on X-gal media (Figure 7E). We found that *attP* segments as small as 44 bp remained competent for *in vivo* recombination, but a 42 bp *attP* was inactive (Figure 7A). These results suggest that while a minimum of 50 bp of DNA is required for *attP* function, specific base sequence information is only required within the 44 bp sequence. In support of this interpretation, *in vitro* deletion reactions with the plasmid containing 44 bp *attP* sites were as efficient as those with 50 bp (data not shown).

The *attB* site was dissected in a similar manner. *In vitro* assays with *attB* duplexes of decreasing length showed that 41 bp was minimally sufficient for recombination, although rates were less than 15% of the longer substrates (Figure 7B,D). *In vivo* deletion assays (Figure 7B), as well as *in vitro* reactions on the deletion plasmid substrates (data not shown), showed that sequence information within a 37 bp region was required. We conclude that a 42 to 44 bp segment is minimally required for full *attB* function with specific base sequence information needed over a 36 to 38 bp region. The length of the *attB* site is thus 6 to 8 bp shorter than *attP*.

### Identification of the crossover site

**Figure 8:**
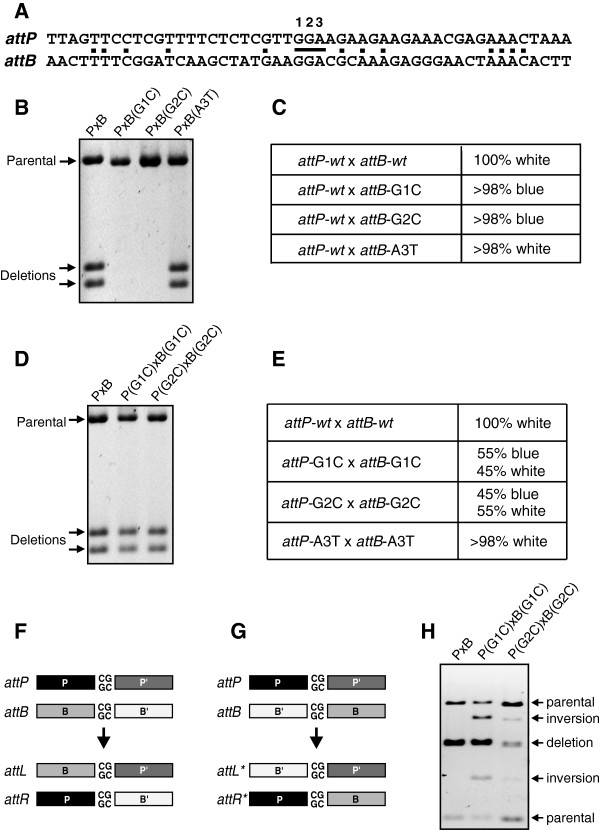
**Determination of the crossover site. (A)** Sequence of the minimal *attP* and *attB* sites, with common nucleotides marked with dots. The central three nucleotides are numbered 1 to 3 in this figure. **(B)***In vitro* intramolecular deletion reactions (30 minutes) on supercoiled plasmids with *attB* sites containing mutations at the central three nucleotides as designated. P×B, wild-type control. The products were digested with *Xho*I and *Bam*H1 to reveal deletion products (Figure 1D). **(C)** Results of *in vivo* deletion reactions between *attP* and mutated *attB* sites. White colonies result from site-specific deletions and blue colonies signify absence of deletions. **(D)***In vitro* intramolecular recombination reactions (30 minutes) with plasmid substrates containing identical changes within *attP* and *attB* at the designated positions. Parental and deletion product bands are denoted. **(E)** Results of *in vivo* recombination reactions between mutated *attP* and *attB* sites. Because G1C and G2C mutations create symmetrical cores, inversions between *att* sites oriented in an antiparallel configuration **(G)** form along with deletions. Inversion products retain the Lac^+^ (blue) phenotype. **(F)** Diagram of synapsis between *attP*(G1C) and *attB*(G1C) in the standard parallel orientation generating *attL* and *attR* upon DNA exchange. Productive recombination between wild-type *attP* and *attB* sites containing the asymmetric GG core nucleotides only occur by this pathway. **(G)** Diagram of an antiparallel synapsis between *attP*(G1C) and *attB*(G1C) generating the hybrid attL* and attR* sites upon DNA exchange. **(H)***In vitro* intramolecular recombination reactions (30 minutes) with plasmid substrates containing identical changes within *attP* and *attB* within the core nucleotides. The reaction products were digested with *Bam*HI, *Nde*I, and *Sca*I to reveal both deletions (doublet bands) and inversions as denoted.

There are only 3 bp in common between the centers of *attP* and *attB* (Figure 7A), implicating the crossover region to be within this segment [1]. To precisely define the crossover site, we individually changed each of these positions in *attB* and evaluated their ability to support intramolecular deletion reactions with *attPwt in vitro* and *in vivo* (Figure 8B,C). Changes at G1 or G2 abolished recombination whereas A3T had no effect on recombination efficiency, implicating the GG dinucleotide as the crossover segment. To confirm this assignment, we made the same changes in *attP* and measured recombination between mutant *attP* and *attB* sites. The double mutants with identical changes at G1 or G2 were active for recombination *in vitro* (Figure 8D) and generated about 50% recombinant transformants (white colonies) *in vivo* (Figure 8E).

### Orientation of *att* sites is distinguished solely by the 2 bp crossover region

In other serine recombinase systems, recombination site orientation is solely determined by the asymmetry of the two core nucleotides at the crossover site. In the case of the G1C or G2C mutants the core nucleotides are symmetrical such that both standard parallel (Figure 8F) as well as antiparallel (Figure 8G) synapses may be productive for recombination. When the products of *in vitro* recombination reactions were analyzed in the double mutants using restriction enzymes whose cleavage distinguishes between inversion and deletion, both product types were present. *attP*(G1C)×*attB*(G1C) generated 64.5% deletion and 35.5% inversion products, and *attP*(G1C)×*attB*(G1C) generated 52.5% deletion and 47.5% inversion products. Sequence analysis of representative plasmids obtained in the *in vivo* experiment in (E) confirmed that the white (*lacZ*^–^) plasmids were the predicted site-specific deletion products and that the blue plasmids contained inversions with hybrid *attL* and *attR* sites as denoted in (G). Recombination sites containing a symmetric dinucleotide core sequence can thus productively synapse in either orientation to generate both deletion and inversion products. The native asymmetric GG dinucleotides must then solely specify directionality of the *att* sites in the A118 integrase system.

## Discussion

In this work we have established and characterized a robust *in vitro attP*×*attB* integration reaction by the A118 integrase. We have used this system to elucidate basic properties of the A118 integrase including its domain structure and binding properties to the recombination sites. We have also defined the DNA sequence boundaries specifying the *attP* and *attB* recombination sites and mapped the 2 bp region where DNA exchange occurs. The sequence of these two core base pairs defines the orientation of the *att* sites. Thus far we find that the A118 integration reaction shares fundamental properties with other characterized reactions by members of the serine recombinase, especially those of the integrase subfamily containing large C-terminal domains.

Partial proteolysis of the A118 integrase by chymotrypsin or proteinase K demonstrated that the full-length enzyme consists of two distinct folded domains, an N-terminal domain corresponding to the catalytic core that is common to all serine recombinases linked to a large domain that constitutes over two-thirds of the protein (Figure 2E). The large C-terminal domain mediates *att* site binding as is observed for other members of this subfamily [24,29,42]. Given the substrate specificity of chymotrypsin, the site for chymotrypsin cleavage is most likely to be at Met136. When compared with available X-ray structures, the protease sensitive site would be within the oligomerization α-helix E segment, shortly past the region that is predicted to be in contact with the catalytic core. A118 integrase Met136 corresponds to γδ resolvase residue 126 that is located in the region of helix E that becomes disordered in the absence of DNA [43]. When bound to DNA, integrase becomes largely resistant to chymotrypsin, consistent with formation of an extended helix, as seen for the γδ resolvase dimer–DNA complex [44]. The segment within the predicted helix E region of integrase, however, remains sensitive to proteinase K activity in the presence of *attP* DNA.

Proteinase K digestion generates an additional fragment that corresponds to cleavages at the C-terminal end of the catalytic core (at or prior to residue 96 corresponding to near the C-terminal end of a putative helix D) and shortly past the basic cysteine/histidine-rich segment within the C-terminal domain (at or shortly beyond residue 331 at the end of a predicted α-helix). Significantly, this fragment is not observed when integrase is bound to *attP*. Potential models – in light of the fact that the C-terminal domain mediates DNA binding – are that bound *attP* DNA directly occludes the protease or induces a conformation change that masks the scission site near residue 331. These would result in the accumulation of a stable C-terminal proteinase K product from approximately residue 136 to the C-terminal end as observed in Figure 2D (lanes 7 and 8). The analogous cysteine-rich segment, together with zinc, has been implicated to be involved in binding DNA by the ϕC31 integrase [32]. Our results are consistent with this region being involved directly or indirectly in DNA binding, but we have not obtained evidence for a role of zinc in DNA binding or recombination by A118 integrase. Most other serine integrases also are reported to function effectively without zinc [27,33-35]. The precise mode of DNA binding remains to be determined for any of the large serine recombinase subfamily members, and data accumulated from several systems suggest there may be significant differences [23].

We found that full-length A118 integrase binds *att* DNA in a highly cooperative manner consistent with it being a dimer in solution. The dimer is even the dominant form bound to half-*att* sites, although binding is poor. On the contrary, the isolated C-terminal domain, which chromatographs as a monomer and exhibits no evidence for multimerization in solution by amine crosslinking (data not shown), binds noncooperatively to *attP* or *attB*. This is different to that observed for the C-terminal domain of ϕC31 integrase, which cooperatively binds to its respective *att* sites even though it is also monomeric in solution [28]. Dimerization by the intact protein appears extremely sensitive to the zwitterionic detergent CHAPS, even more sensitive than the Hin invertase. The effect of CHAPS on *att* binding, particularly at *attB* where both half-sites exhibit low affinity, is mirrored by its inhibitory effect on recombination. The sensitivity to CHAPS, together with the modest stimulation by ethylene glycol and glycerol, suggests that fundamental features of the dimer to tetramer transition involving the oligomerization helix that presumably accompany formation of integrase synaptic complexes are likely to share similarities with Hin [10,40,41,45].

The A118 integrase only efficiently supports recombination between *attP* and *attB*, and this reaction is relatively efficient regardless of whether the sites are located on separate DNA molecules or in cis (either in an inverted or directly repeated orientation). Surprisingly, a low efficiency reaction between two *attP* sites was observed *in vitro*, but importantly with respect to the phage lifecycle no reactions involving *attL* or *attR* sites were detected with integrase alone. Binding of integrase to each of the four *att* sites is similar and thus cannot explain the site selectivity for recombination. We have recently identified an additional phage-encoded protein that is required for the excisive *attL*×*attR* reaction, which will be the subject of a future report. Other serine integrases also exhibit exquisite site-selectivity for recombination [24,26,27,33] and have been found to require recombination directionality factors or Xis-like proteins for *attL*×*attR* recombination [27,46-48]. Mechanisms underlying site-selective synapsis are a major area of exploration for the integrase subfamily of serine recombinases.

As is the case for most of the other serine integrases [23], the sequences defining the A118 *attP* and *attB* sites are remarkably unrelated (Figure 7). Unlike most of the other characterized systems, the minimal A118 *attB* site exhibits essentially no symmetry. The A118 *attP* site requires specific sequences extending 22 bp on either side of the 2 bp crossover site, and *attB* requires about 19 bp. An additional 3 bp of flanking DNA is required for minimal *att* function, presumably reflecting DNA backbone contacts needed for A118 integrase binding. Whereas minimal *att* site lengths vary somewhat between systems, most require between 35 and 55 bp. Similar to A118, many integrases such as Bxb1, ϕC31, ϕBT1, ϕRv1, TG1, and TP901 [24,27,33,34,49,50], but not all (for example, R4 [51]), require a longer sequence at *attP*. Both full-length and the isolated C-terminal domain of A118 integrase interact with *attP* and *attB* asymmetrically, with the *attP* and *attB* left half-sites exhibiting much higher affinity than the right half-sites. Whether this difference is functionally important is not known, but a symmetrical *attB* (BoB) containing two good half-sites was a poor recombination substrate (data not shown). The sequence asymmetry cannot be a determinant of orientation-dependent *att* site synapsis since sites with symmetric core base pairs efficiently recombined from both parallel and antiparallel synapses (Figure 8E,F,G,H). The ϕC31 *attB* and Bxb1 *attP* sites have also been reported to bind to their respective integrases in an asymmetric manner [24,52].

Finally we note that the *in vitro* A118 *attP*×*attB* reaction is efficient under a variety of substrate topology and solution conditions. The most efficient reaction occurs when the *att* sites are located in cis on a supercoiled molecule in the presence of spermidine and divalent cations where rates up to three recombinants per minute are obtained. However, the reaction occurs readily in the presence of metal chelators and without a polyamine and on linear DNA. These properties are generally similar to other serine integrases but contrast with members of the resolvase/invertase subfamily [17,18]. A mechanistically interesting feature of the A118 integrase deletion reaction is the formation of topologically unlinked circular deletion products, even when the *att* sites are separated by many kilobases on a supercoiled DNA molecule (unpublished data). The abundance of free deletion products implies constraints on the assembly of the synaptic complex and the subunit rotation reaction.

## Conclusions

There are a number of fundamental questions regarding the mechanism of recombination reactions catalyzed by serine integrases, particularly with respect to the roles of their C-terminal domains, whose sizes dwarf the much smaller catalytic domains. These include, but are not limited to, mechanisms underlying the exquisite site selectivity for productive synapsis, and the architecture of the active recombination complex. In this report we have characterized the biochemical properties and substrate requirements of the *attP*×*attB* integration reaction by the phage A118 integrase. The reaction is robust and amenable to more detailed studies on its reaction mechanism. The A118 recombinase thus joins the handful of model serine integrase systems where *in vitro* studies are advancing our understanding of this subfamily. Studies on members from the different subclasses (Figure 1B) will almost certainly reveal both unique and common features of the subfamily. Information from the different systems will be essential for a full understanding of how these enzymes function and how they can be utilized to their fullest potential for genetic engineering.

## Methods

### Strains, phages and recombinant plasmids

A list of plasmids used in this work is presented in Table 1. Richard Calendar (University of California – Berkeley) kindly provided the following *L*. *monocytogenes* strains and DNA: DP-L3689 (strain 10403 lysogenized with A118), DP-L3670 (strain 10403S lysogenized with U153), DP-L4056 (strain 10403S cured of phage), and phages A118 and U153. The A118 integrase coding sequence was amplified by PCR from the phage DNA and cloned between *Nde*I and *Bam*HI sites of pET11a and pET15b (EMD Millipore Billerica, MA USA) to give pRJ2186 and pRJ2184, respectively. pRJ2823 contains integrase residues 158 to 452 (C-terminus) in pET15b. The *att* sites were initially cloned into pBR322 with 100 or 200 bp of native sequence flanking on each side as PCR fragments using DNA from the phage, lysogen, or cured strain.

**Table 1:** Plasmids used in the present study

**Plasmid**	**Description**^**a**^	**Source**
pRJ2184	pET15b, A118 integrase between *Nde*I-*Bam*H1	This work
pRJ2186	pET11a, A118 integrase between *Nde*I-*Bam*H1	This work
pRJ2823	pET15b, A118 integrase^158-452^ between *Nde*I-*Bam*H1	This work
pRJ2214	pBR322, *attP* (400 bp) into *Eco*R1	This work
pRJ2215	pBR322, *attB* (200 bp) into *Sal*I	This work
pRJ2289	pBR322, *attP*^U153^ (200 bp) into *Eco*R1	This work
pBCPB+	pBCSK+, *cam* (*lacZ*)	Groth and colleagues [50]
pIntA1	pACYC177, *kan lacP*-A118 integrase	Keravala and colleagues [31]
pBCPB-A1+	pBCSK+, *cam* DR(*attB*-*lacZ*-*attP*)	Keravala and colleagues [31]
pRJ2799	pBCPB+, *cam* IR(*attB*-*lacZ*-*attP*)	This work
pRJ2825	pBCPB+, *cam* DR(*attB*(G1C)-*lacZ*-*attP*)	This work
pRJ2826	pRJ2825, *attP* (G1C)	This work
pRJ2827	pBCPB+, *cam* DR(*attB*(G2C)-*lacZ*-*attP*)	This work
pRJ2828	pRJ2827, *attP* (G2C)	This work
pRJ2829	pBCPB+, *cam* DR(*attB*(A3T)-*lacZ*-*attP*)	This work
pRJ2830	pRJ2829, *attP* (A3T)	This work
pRJ2831	pBCPB+, *cam* DR(*attB*^44^-*lacZ*-*attP*)	This work
pRJ2927	pBCPB+, *cam* DR(*attB*^41^-*lacZ*-*attP*)	This work
pRJ2832	pBCPB+, *cam* DR(*attB*^39^-*lacZ*-*attP*)	This work
pRJ2833	pBCPB+, *cam* DR(*attB*^37^-*lacZ*-*attP*)	This work
pRJ2834	pBCPB+, *cam* DR(*attB*^35^-*lacZ*-*attP*)	This work
pRJ2835	pBCPB+, *cam* DR(*attB*-*lacZ*-*attP*^50^)	This work
pRJ2836	pBCPB+, *cam* DR(*attB*-*lacZ*-*attP*^*48*^)	This work
pRJ2848	pBCPB+, *cam* DR(*attB*-*lacZ*-*attP*^46^)	This work
pRJ2837	pBCPB+, *cam* DR(*attB*-*lacZ*-*attP*^44^)	This work
pRJ2928	pBCPB+, *cam* DR(*attB*-*lacZ*-*attP*^42^)	This work
pRJ2913	pBCPB+, *cam* DR(*attL*-*lacZ*-*attR*)	This work
**Strain**		
DP-L 3689 (10403::A118)		R Calendar
DP-L 3670 (10403S::U153)		R Calendar
DP-L 4056 (10403S phage cured)		R Calendar

^a^*att* sites are from A118 unless specified. DR, *att* sites in direct orientation; IR, *att* sites in inverted orientation.

Substrates for *in vivo* recombination assays were derived from pBCPB-A1+, which was provided by Michele Calos (Stanford University, CA, USA). pBCPB-A1+ is a colE1-based plasmid that contains the *lacZ* gene flanked in direct repeat orientation with 200 bp *attB* (between *Bam*HI and *Xho*I sites) and *attP* (*Sma*I site) segments [31]. A deletion reaction results in 3.0 plus 3.4 kb product circles (Figure 3D). pRJ2799 contains the *attP* and *attB* sites in inverted orientation relative to each other and was constructed by substituting 200 bp A118 *attP* and *attB* sites into *Sma*I and *Bam*HI-*Xho*I sites, respectively, in pBCPB+ [51]. pRJ2913 contains *attR* and *attL* in direct repeat orientation and was constructed in a similar manner as described for pRJ2799. Integrase for *in vivo* assays was supplied by pInt that has the A118 integrase controlled by *lacP* on a p15A origin plasmid [31]. For minimal *att* site length determination, different length *attP* or *attB* duplex oligonucleotides were substituted for their respective sites in pBCPB-A1+. Individual base pair substitutions within *att* sites were generated by QuikChange mutagenesis.

### Integrase purification

RJ3386 (BL21(DE3) *endA*::Tn*10*) containing His-tagged integrase overexpressing plasmids were grown in 1 l LB with ampicillin (100 μg/ml) at 37°C until the optical density at 600 nm reached 0.6. The cultures were cooled to 10°C, isopropylthio-β-galactoside added to 0.5 mM, and incubation continued for 18 to 20 hours at 10°C with shaking. Harvested cells were resuspended in 20 ml lysis buffer (20 mM HEPES, pH 7.5, 200 mM NaCl, 20 mM imidazole, 1 mM DTT, 10% glycerol, and 0.1 mM phenylmethanesulfonyl fluoride and lysed by two passes through a French press. The lysate was centrifuged at 26,000×*g* for 30 minutes, and the supernatant loaded onto a 1 ml Ni-NTA column that was then washed with 20 mM HEPES, pH 7.5, 0.5 M NaCl, 40 mM imidazole and 10% glycerol, and bound protein eluted with the same buffer but with 0.3 M NaCl and 200 mM imidazole. Full-length ^His^Int was then loaded directly onto a column containing 0.5 ml Heparin Sepharose 6 Fast Flow (GE Healthcare Biosciences, Pittsburgh, PA USA) column, washed with 20 mM HEPES, pH 7.5, 0.5 M NaCl, 1 mM DTT, and 10% glycerol, and eluted with the same with 1 M NaCl. Native integrase was obtained by heparin-Sepharose chromatography essentially as above. Integrase preparations were stored in 20 mM HEPES, pH 7.5, 1 M NaCl, 1 mM DTT, 0.1 mM EDTA and 40% glycerol at −20°C.

Size exclusion chromatography was performed through an FPLC Superdex-200 10/300 GL column (GE Healthcare Biosciences, Pittsburgh, PA USA) in 20 mM HEPES, pH 7.5, 1 M NaCl, 1 mM DTT, and 10% glycerol at a flow rate of 0.4 ml/minute at 23°C.

### *In vitro* recombination reactions

Standard recombination reactions were typically performed in a buffer containing 20 mM HEPES, pH 7.5, 100 mM NaCl or KCl, 5 mM spermidine, 2.5 mM DTT, 5 mM CaCl_2_ or MgCl_2_, 30 μg/ml BSA, and 5% glycerol and incubation was at 30°C. Intramolecular deletion and inversion reactions were performed with 0.02 pmol plasmid DNA and intermolecular integration reactions typically utilized 0.03 pmol supercoiled plasmid DNA and 0.09 pmol linear DNA (50 to 100 bp fragment derived from PCR or synthetic duplex oligonucleotides). Reactions were initiated by addition of 0.5 to 1 pmol purified integrase and terminated by inactivation at 65°C or addition of 1% SDS. Intramolecular recombination reactions were digested with *Bam*HI and *Xho*I restriction enzymes to generate linear deletion products or reveal inversion prior to agarose gel electrophoresis.

### *In vitro* DNA binding assays

DNA probes were prepared by polynucleotide kinase reactions with γ-^32^P-ATP of gel-purified PCR-generated fragments or oligonucleotides. Varying concentrations of integrase were added to approximately 0.1 pmol ^32^P-DNA in a buffer containing 20 mM HEPES, pH 7.5, 100 mM NaCl, 0.1 mg/ml BSA, 5 mM spermidine, 5 mM DTT, 5% glycerol, and 50 μg/ml sonicated salmon sperm DNA in a total volume of 10 μl. The reactions were incubated at 30°C for 30 minutes and the protein–DNA complexes were separated on nondenaturing 6% polyacrylamide gels in 0.5× TBE buffer at room temperature. Gels were dried and analyzed by phosphorimaging.

### *In vivo* recombination assays

Recombination substrate plasmids (Cam^r^) were transformed into *E*. *coli* DH5α containing pIntA1 (Kan^r^) and plated on LB agar plates containing chloramphenicol, kanamycin, and X-gal (20 μg/ml). After overnight incubation at 37°C, blue (unrecombined) and white (deletion) colonies were scored.

### Partial proteolysis and mass spectrometry

Different amounts of chymotrypsin (Sigma-Aldrich, St. Louis, MO USA) and proteinase K (Sigma-Aldrich) were added to 3 μg integrase in 20 μl PBS and incubated at 23°C for 10 minutes. 4-(2-Aminoethyl) benzenesulfonyl fluoride hydrochloride (2.5 mg/ml) and phenylmethanesulfonyl fluoride (2 mM) were added to terminate the chymotrypsin and proteinase K reactions, respectively. SDS sample buffer was then added, the samples were heated at 95°C for 10 minutes, and proteolysis products were analyzed by 15% SDS-PAGE. For partial proteolysis of integrase in the presence of DNA, integrase was incubated with a twofold molar excess of *attP* (50 bp) oligonucleotides at 23°C for 10 minutes before digestion. For mass spectrometry, gel fragments were excised, in-gel digested with trypsin (Trypsin Gold, Mass Spectrometry Grade; Promega Madison, WI USA), and analyzed using positive reflector mode on a MALDI-TOF/TOF Ultraflex instrument (Bruker Daltonics Inc., Billerica, MA, USA). Peptides were identified using the Mascot Server software v.2.2 (Matrix Science Ltd., London, UK).

## Abbreviations

Bp: Base pair; BSA: Bovine serum albumin; CHAPS: 3-((3-cholamidopropyl)dimethylammonio)-1-propanesulfonate; DTT: Dithiothreitol; EDTA: Ethylenediamine tetraacetic acid; K_d_: Dissociation constant; LB: Luria Bertani broth; MALDI-TOF/TOF: Matrix-assisted laser desorption/ionization time of flight/time of flight; PBS: Phosphate-buffered saline; PCR: Polymerase chain reaction; SSR: Site-specific recombination.

## Competing interests

The authors declare that they have no competing interests.

## Authors’ contributions

SM generated most of the data in the manuscript. GD initiated work on the project and generated early data. NKA and MJH performed the mass spectrometry. SM, GD, and RCJ participated in the design of the study. RCJ and SM drafted the manuscript. All authors read and approved the final manuscript.

## Authors’ information

Joint first author: Sridhar Mandali and Gautam Dhar.
